# Sun protection behaviors among children aged 6−18 years old, the role of socioeconomic factors: A cross‐sectional study

**DOI:** 10.1002/hsr2.1727

**Published:** 2023-11-21

**Authors:** Nikta Nouri, Parisa Iravani, Bahareh Abtahi‐Naeini

**Affiliations:** ^1^ School of Medicine Isfahan University of Medical Sciences Isfahan Iran; ^2^ Pediatrics Department, School of Medicine Isfahan University of Medical Sciences Isfahan Iran; ^3^ Pediatric Dermatology Division of Pediatric Department, Imam Hossein Children's Hospital Isfahan University of Medical Sciences Isfahan Iran; ^4^ Skin Diseases and Leishmaniasis Research Center Isfahan University of Medical Sciences Isfahan Iran

**Keywords:** children, risk reduction behaviors, skin cancer, sunlight, sunscreens, ultraviolet light

## Abstract

**Background and Aims:**

Evaluation of sun protection behavior and related factors in children aged between 6 and 18 years in Isfahan, Iran.

**Methods:**

This cross‐sectional study was conducted at outpatient pediatric clinics affiliated with Isfahan University of Medical Sciences, Isfahan, Iran during the summer of 2021. A checklist was designed, and the interviewer used that to gather the required information including children's demographic characteristics, families' socioeconomic data, and sun‐protective behaviors in children.

**Results:**

The mean ± standard deviation (SD) age of children was 10.54 ± 3.61. Among the study population, 63.7% of children were male. The most common skin phototypes were II and III (33.5% each). 83.8% of children used at least one method of sun protection (94.5% of girls, 77.6% of boys, *p* < 0.001). Wearing long‐sleeved clothes was the most frequent UV‐protection method (48%), while sunscreen application was the least prevalent method (28.1%). Sun protection behaviors were more frequent among urban children (86.7%, *p* = 0.009) and children with wealthy families (94%, *p* = 0.035). Sun protection methods were used by most of the children whose mothers applied sunscreen on a daily basis (90.2%, *p* = 0.002) and all of the children whose mothers held a master's degree or above (100%, *p* = 0.004).

**Conclusion:**

Children's sun protection behavior is directly associated with demographic characteristics, families' socioeconomic level and maternal usage of sun protection measures. It is necessary to provide information and education about sun protection methods and the risks of excessive sun exposure to families and children, as well as facilitate their access to these.

## 1 INTRODUCTION

Solar ultraviolet (UV) radiation is considered to be the primary cause of skin cancers.^1^ As a result of constant exposure to sunlight and solar UV radiation deoxyribonucleic acid (DNA) damage can occur, leading to photoaging and cancer.^2^, ^3^ This results from the interaction between UV light and a subset of molecules called chromophores. The absorption of UV light by chromophores in the epidermis results in adverse biological effects including oxidative damage, activating transcription factors, DNA mutations, and damage to cell membranes.^4^

Recently there is an increasing trend in skin cancer incidence worldwide.^5^ In the United States, skin cancer affects one in five people.^6^ In Iran, skin cancer accounts for 32.7% of all cancers diagnosed.^7^ Skin cancer mortality, in Iran, has grown around ten‐fold between 1995 and 2004 (from 0.06 to 0.7 in 100,000 people), despite being preventable.^8^ There are some known risk factors for skin cancers which could be divided into endogenous and exogenous factors.^9^ Endogenous factors include skin phototype, presence and number of melanocytic nevi, and familial history of skin cancer.^9^ Cumulative sun exposure duration, UV protection behaviors, and history of sunburn are among exogenous factors.^10^ Excessive UV radiation is reported to be the most significant risk factor for skin cancer, accounting for over 90% of nonmalignant skin cancer and 65% of malignant melanoma globally.^11^, ^12^

It has been shown that 25−50% of the total UV exposure up to the age of 60 is accumulated during childhood, and children have up to three times higher annual sun exposure compared to adults.^13^ Given that children's skin, especially that of those under the age of three, has less melanin and a thinner stratum corneum and therefore making it more sensitive to UV radiation,^14^, ^15^ it can be deduced that early exposure to sunlight significantly affects the incidence of skin cancer in the future.^12^ Thus, both primary (photoprotection) and secondary prevention should be applied at an early stage of life.^16^, ^17^ Based on the protocol published by World Health Organization (WHO), photoprotection is achieved by avoiding the sun, staying in shade, wearing clothes that block the sunlight, hats, and sunglasses, and using sunscreens.^18^, ^19^

Despite the high incidence of skin cancer in Iran,^20^ there are limited studies regarding sun protection behaviors in the Iranian population, especially among children. In addition, due to the importance of sunburn prevention and sunlight protection in childhood, as significant factors in reducing the risk of skin cancer,^21^ studies regarding the usage frequency of sun protection methods and the variables affecting sun protection behaviors are crucial. In the current study, we investigate the frequency of sun protection behaviors among children and their mothers in Isfahan, a city located in the center of Iran. We also evaluate the association between demographic/socioeconomic variables and sun protection behaviors in children.

## 2 MATERIALS AND METHODS

### 2.1 Study setting and selection criteria

This cross‐sectional descriptive study was conducted at outpatient pediatric clinics in two different regions of the city (Imam Hossein Children's Hospital and Al‐Zahra Hospital), affiliated with Isfahan University of Medical Sciences, Isfahan, Iran during the summer of 2021. To calculate the sample size while considering a 95% confidence level ($z_{\alpha }=1.96$), sun‐protective behavior relative frequency of 68.8% (p=0.688), according to the study by Nahar et al.^22^ and a margin of error of 0.06 (d=0.06), a sample size (n) of 230 individuals was calculated using $n=\left(z_{\alpha }^{2}\;p\;\left(1-p\right)\right)/d^{2}$formula. The ethical committee of Isfahan University of Medical Sciences approved this study (Ethical code: IR.MUI.MED.REC.1400.012) in accordance with the latest version of the Helsinki Declaration. All participants received a detailed description of the study, and those who consented were interviewed; those who did not consent were exempted. Mothers were interviewed regarding their children's sun protection behaviors. Adolescents cooperated in providing their information to the interviewers as well.

The inclusion criteria for case selection were (i) children aged between 6 and 18 years, and (ii) sufficient knowledge of the Persian language. The exclusion criteria were (i) inherited photodermatosis/genodermatosis and (ii) incomplete information.

### 2.2 Data collection

A checklist was designed including the following information: children's gender, age, skin phototype (based on the Fitzpatrick scale of skin phototypes^23^), underlying diseases (divided into dermatologic and nondermatologic subgroups), usage of sun protection methods (sunscreen, wearing long‐sleeved clothes, sunglasses, wide‐brimmed hat, and visor) in the last month, daily sunscreen renewal in the last month, mean daily sun exposure in the last month, and sunburn history; mother's age, educational level (including six categories: no education, elementary school, undergraduate, associate's degree, bachelor's degree, and master's degree or above), employment, and daily sunscreen use during the last month; family's place of residence (urban/rural), and monthly income (divided into three categories: less than 50 M IRR, 50−100 M IRR, and more than 100 M IRR). Through the written checklist, the interviewer collected the required data. Data were gathered from two geographically distinct hospitals to reduce the potential biases.

### 2.3 Statistical analysis

Variables that were continuous were expressed as means with standard deviations (SD), while categorical variables were expressed as numbers (percentages). We compared the means of the continuous variables between two or more groups, using the independent Student *t*‐test (or analysis of variance test) and the Mann−Whitney test (or Kruskal−Wallis test) for the variables with normal and non‐normal distributions, respectively. We assess the difference in the distribution of the categorical variables between groups of demographic variables using the *χ* ^2^ test. We used SPSS 20.0 for Windows to conduct statistical analyses. The statistical significance of this study was determined by the two tailed *p* Values less than 0.05.

## 3 RESULTS

Among a total of 373 enrolled children, 21 of them had incomplete data and were excluded from the study. The mean ± SD age of children and their mothers were 10.54 ± 3.61 and 36.6 ± 5.9 respectively. 63.7% of children were male. Children's/mothers' characteristics are summarized in Table 1 (Table 1). The most common skin phototypes were II and III (33.5% each). 17.62% had non‐dermatological underlying diseases while only 0.85% reported dermatological conditions. 69.2% of the families were living in urban areas.

**Table 1:** The demographic, phenotypic, and socioeconomic characteristics of the study population.

Variables	Total number	Mean (SD) or *N* (%)
*Children*		
Age		10.54 (3.61)
*Gender*	350	
Female		127 (36.3)
Male		223 (63.7)
*Skin phototype*	352	
I		44 (12.5)
II		118 (33.5)
III		118 (33.5)
IV		46 (13.1)
V		20 (5.7)
VI		6 (1.7)
*Underlying disease*	352	
None		287 (81.53)
Dermatologic		3 (0.85)
Nondermatologic		62 (17.62)
Mothers		
Age		36.60 (5.960)
*Employment*	330	
Yes		78 (23.6)
No		252 (76.4)
*Education*	339	
No education		21 (6.2)
Elementary school		32 (9.4)
Undergraduate		143 (42.2)
Associate's degree		42 (12.4)
Bachelor's degree		79 (23.3)
Master's degree or above		22 (6.5)
*Living area*	305	
Rural		94 (30.8)
Urban		211 (69.2)
*Family income*	249	
Less than 50 M IRR		45 (18.1)
50−100 M IRR		104 (41.8)
More than 100 M IRR		100 (40.2)

Abbreviations: *N*, number; SD, standard deviation.

83.8% of children used at least one method of sun protection. 94.5% of girls and 77.6% of boys used sun protection methods (*p* < 0.001). Urban children used sun protection methods more than children living in rural areas (86.7% compared to 74.5%, *p* = 0.009). For families with income over 100 M IRR, 94% of their children used sun protection methods, compared to 85.6% among families with 50−100 M IRR income, and 80% among those earning less than 50 M IRR (*p* = 0.035). The frequency of sun protection behaviors among children whose mothers used sunscreen was 90.2% (*p* = 0.002). The percentage of using sun protection methods in children whose mothers held master's degrees and above was 100% (*p* = 0.004). There are no significant correlations between the frequency distribution of children's skin phototypes, the mean age of children/mothers, and sun protection behaviors (*p* > 0.05). Sunburn history was more frequently reported for skin phototypes of I (56.2%) and II (61.1%) as compared to other phototypes (*p* = 0.001).

Table 2 shows the prevalence of the photoprotection methods among children (separately for each method) and correlations between the demographic/socioeconomic characteristics and the various sun protection methods (Table 2). Wearing long‐sleeved clothes (48%) was the most frequent UV‐protection method, while the application of sunscreen was the least used method (28.1%). Moreover, wide‐brimmed hats, sunglasses, and visors were worn by 43.5%, 33%, and 30.1% of the study population, respectively. Furthermore, girls wore long‐sleeved clothes (77%, *p*< 0.001), sunglasses (40.9%, *p* = 0.02), visors (40.2%, *p* = 0.003), and sunscreen (39.4%, *p* = 0.001) significantly more than boys whereas boys used wide‐brimmed hats (52.9%, *p* < 0.001) more. Children living in urban areas wore wide‐brimmed hats (50.2%, *p* < 0.001), sunglasses (43.1%, *p* < 0.001), visors (35.5%, *p* = 0.001), and sunscreens (30.8%, *p* = 0.003) significantly more than rural children. Children from wealthy families (whose income is more than 100 M IRR per month) used sunglasses (60%, *p* > 0.001), sunscreens (49%, *p* > 0.001), and visors (44.4%, *p* = 0.005) more often than children from lower‐income families. Higher prevalence of wearing visors (63.6%, *p* < 0.001), sunglasses (63.6%, *p* < 0.001), wide‐brimmed hats (54.5%, *p* = 0.02), and sunscreen (63.6%, *p* < 0.001) was observed among children whose mothers had higher educational degrees. Compared with other children, children of employed mothers used sunglasses (50%, *p* = 0.001), sunscreens (43.6%, *p* = 0.001), and visors (41%, *p* = 0.02) more frequently.

**Table 2:** Prevalence of photoprotection methods among the study population during the month before the interview.

	Wide‐brimmed hat		Visor		Sunglasses		Long‐sleeved clothes		Sunscreen use	
	*N* (%)	*p*	*N* (%)	*p*	*N* (%)	*p*	*N* (%)	*p*	*N* (%)	*p*
Overall	153 (43.5)	‐	106 (30.1)	‐	116 (33)	‐	169 (48)	‐	99 (28.1)	‐
*Gender*										
Female	33 (26.0)	<0.001	51 (40.2)	0.003	52 (40.9)	0.02	97 (77.0)	<0.001	50 (39.4)	0.001
Male	118 (52.9)	‐	55 (24.9)	‐	64 (28.7)	‐	71 (31.8)	‐	49 (22.0)	‐
*Mother's occupation*										
Employed	37 (47.4)	0.44	32 (41.0)	0.02	39 (50.0)	0.001	36 (46.2)	0.66	34 (43.6)	0.001
Unemployed	107 (42.5)	‐	68 (27.2)	‐	74 (29.4)	‐	123 (49.0)	‐	62 (24.6)	‐
*Mother's educational status*										
No education	4 (19.0)	0.02	1 (4.8)	<0.001	2 (9.5)	<0.001	11 (52.4)	0.29	0 (0.0)	‐
Elementary school	11 (34.4)	‐	6 (19.4)	‐	8 (25.0)	‐	17 (53.1)	‐	5 (15.6)	<0.001
Undergraduate	62 (43.4)	‐	41 (28.9)	‐	33 (23.1)	‐	67 (46.9)	‐	29 (20.3)	‐
Associate's degree	15 (35.7)	‐	10 (23.8)	‐	18 (42.9)	‐	23 (56.1)	‐	19 (45.2)	‐
Bachelor's degree	45 (57.0)	‐	32 (40.5)	‐	39 (49.4)	‐	31 (39.2)	‐	30 (38.0)	‐
Master's degree or above	12 (54.5)	‐	14 (63.6)	‐	14 (63.6)	‐	14 (63.6)	‐	14 (63.6)	‐
*Living area*										
Rural	24 (25.5)	<0.001	15 (16.1)	0.001	11 (11.7)	<0.001	50 (53.2)	0.15	14 (14.9)	0.003
Urban	106 (50.2)	‐	75 (35.5)	‐	91 (43.1)	‐	93 (44.3)	‐	65 (30.8)	‐
*Family income*										
Less than 50M IRR	19 (42.2)	0.55	12 (26.7)	0.005	6 (13.3)	<0.001	20 (44.4)	0.85	6 (13.3)	<0.001
50−100 M IRR	50 (48.1)	‐	25 (24.0)	‐	31 (29.8)	‐	49 (47.1)	‐	22 (21.2)	‐
More than 100 M IRR	52 (52.0)	‐	44 (44.4)	‐	60 (60.0)	‐	49 (49.5)	‐	49 (49.0)	‐

Abbreviation: *N*, number.

Table 3 presents the relationship between demographic characteristics and frequency distributions of sunburn history, daily sunscreen renewal, the mean daily sun exposure in the previous month for children, and mothers' sunscreen use (Table 3). Mean daily sun exposure in the last month for boys (*p* < 0.001) and urban children (*p* = 0.002) was significantly higher than for girls and rural children, respectively. Mothers with higher educational levels (more than 67%, *p* < 0.001) and employed mothers (78.2%, *p* < 0.001) had more frequent sunscreen daily use.

**Table 3:** Prevalence of sunburn history, children and mothers' sunscreen use, and sun exposure during the month before the interview.

	History of sunburn		Children's sunscreen renewal		Mothers' daily sunscreen use		Daily sun exposure		
	*N* (%)	*p*	*N* (%)	*p*	*N* (%)	*p*	Median (IQR)	Mean (SD)	*p*
Overall	161 (45.7)	‐	31 (8.8)	‐	183 (52)	‐	2 (1−3)	2.48 (1.95)	‐
*Gender*									
Female	65 (53.3)	0.09	15 (37.5)	0.79	‐	‐	1.50 (1−2.75)	1.88 (1.46)	<0.001
Male	96 (43.8)	‐	16 (34.8)	‐	‐	‐	2 (1−4)	2.81 (2.12)	‐
*Mother's employment*									
Employed	38 (48.7)	0.81	10 (37.0)	0.82	61 (78.2)	<0.001	2 (1−3)	2.31 (1.82)	0.49
Unemployed	115 (47.1)	‐	19 (34.5)	‐	119 (47.2)	‐	2 (0.94−2.06)	2.49 (1.93)	‐
*Mother's educational status*									
No education	7 (35.0)	0.43	0	‐	2 (9.5)	<0.001	2 (1−4)	2.88 (2.87)	0.24
Elementary school	15 (48.4)	‐	3 (75.0)	0.44	9 (28.1)	‐	3 (1−4)	3.16 (2.24)	‐
Undergraduate	59 (42.8)	‐	7 (29.2)	‐	68 (47.6)	‐	2 (1−3)	2.30 (1.88)	‐
Associate's degree	21 (51.2)	‐	5 (29.4)	‐	31 (73.8)	‐	2 (1−4)	2.68 (1.87)	‐
Bachelor's degree	44 (55.7)	‐	11 (40.7)	‐	53 (67.1)	‐	2 (1−3)	2.45 (1.92)	‐
Master's degree or above	10 (45.5)	‐	4 (33.3)	‐	19 (86.4)	‐	1.75 (1−3)	2.11 (1.47)	‐
*Living area*									
Rural	37 (43.5)	0.26	4 (28.6)	0.35	32 (36.8)	0.001	2 (1−3)	1.99 (1.37)	0.002
Urban	107 (50.7)	‐	22 (42.3)	‐	123 (58.3)	‐	2 (1−4)	2.75 (2.09)	‐
*Family income*									
Less than 50 M IRR	15 (33.3)	0.005	0 (0.0)	0.10	11 (24.4)	<0.001	2 (1−3.25)	2.28 (1.91)	0.45
50−100 M IRR	47 (45.2)	‐	5 (25.0)	‐	50 (48.1)	‐	2 (1−3)	2.43 (1.92)	‐
More than 100 M IRR	61 (61.0)	‐	19 (42.2)	‐	76 (76.0)	‐	2 (1−3)	2.54 (1.72)	‐

Abbreviations: IQR, interquartile range; *N*, number; SD, standard deviation.

## 4 DISCUSSION

The result of our study demonstrated that about 80% of children used at least one method of sun protection, while applying sunscreen was the least frequent method. We found that the most frequent sun protection behaviors were wearing long‐sleeve clothes and wide‐brimmed hat. Sun protection behaviors and sunburn history were strongly correlated with demographic, phenotypic, and socioeconomic characteristics in children. There are some differences in sun protection behaviors between the two genders. Among girls, the prevalence of wearing long‐sleeved clothes, sunglasses, and visors was higher while wearing wide‐brimmed hats was more common among boys. Children whose mothers were employed, had higher education, came from wealthy families, and lived in urban areas were more likely to use sunscreen and other sun‐protective methods, such as sunglasses, visors, and wide‐brimmed hats.

In our study population, the most common sun‐protective method among children was wearing long‐sleeve clothes. This result is in agreement with the previous studies in Iran.^24^, ^25^ Our study found that sunscreen was the least commonly used method. In a similar study in southwest Iran, among 385 high‐school students, wearing long‐sleeve clothes was the most common sun protection method, and applying sunscreen was the second most prevalent.^24^ The discrepancy in the prevalence of sunscreen use could be due to the difference between the mean age of the participants in our study (about 10 years) and those in the previous study (about 14 years). It is possible that younger children are less inclined to use sunscreen. Nevertheless, the findings reported by Patel et al. contradict this notion, as they found that the prevalence of sunscreen use was higher among younger children.^26^ This suggests that sun protection behavior is influenced by multiple factors. Also, it is expected that the use of each sun protection method varies across different regions. Comparing two studies conducted in the United States, wearing long‐sleeve clothes was the most prevalent sun protection method in Minnesota (about 70%), while it was the least common method in Florida (less than 10%).^27^, ^28^

In a telephone survey in Australia, among 1140 parents/guardians of children aged under 11 years old, the two most commonly used methods were wearing hats and sunscreen.^29^ The differences in the prevalence of sun protection methods between our study and the one in Australia could be related to the higher public awareness regarding sun protection methods compared to Iran. Public educational programs regarding skin cancer prevention have been implemented in Australia for decades.^29^

In our study, there are some significant differences in sun protection behaviors as well as a difference in mean daily sun exposure between boys and girls. In agreement with our study, similar results were reported in the study conducted among 860 children in three regions of the United States.^26^ Possible explanations for this could be the differences in cultural and mental norms as well as the differences in gender‐specific clothing. Moreover, the Hijab in Iran might be also a reason for the higher prevalence of wearing long‐sleeve clothes among females. Furthermore, girls tend to care more about cosmetics and beauty; which can result in more frequent utilization of sunscreen. In line with our findings, in a previous study in Iran among 10,555 high‐school students, the scores of knowledge, attitude, and behavior in sun protection were higher among females.^25^

Results of our study demonstrated, there is a significant correlation between mothers' sunscreen use and their children's sun protection behaviors. This can be due to children viewing their mothers as role models when it comes to sun protection.^30^, ^31^ Furthermore, children whose mothers held higher educational degrees or were employed exhibited more frequent sun‐protective behaviors. Similar findings were achieved in previous studies by other research groups.^27^, ^32^ It is possible that higher educational levels are associated with health awareness among mothers. According to a previous study, students whose parents held university degrees exhibited higher knowledge and practice scores regarding sun‐protective measures.^33^ The use of sunglasses and sunscreen was more prevalent in higher socioeconomic families. This may be attributed to the higher cost barrier of these methods as well as the increased financial resources available to high‐income families. Similar to our results, a study by Robinson et al. among 503 households in the United States, reported more frequent sunscreen usage among children in families with high annual incomes.^34^

The use of some sun protection measures in urban children was significantly more than children in rural areas, which can be due to easier access to various educational resources; for example, health educational centers, dermatology offices, and clinics. On the one hand, continuous education and intervention can help to improve sun protection behaviors.^35^, ^36^ On the other hand, cultural and geographical factors can also affect sun protection behaviors. Our results are in line with the findings of the study conducted in urban and rural areas of Utah in the United States among high‐school students. This study demonstrated that the sun protective behaviors were less practiced among rural students compared to the ones living in urban areas, showing the significance of geographical factors impacting the sun‐protective behaviors of individuals.^37^ The study by Bolick et al. conducted in different regions of the United States found that geographical location can have a slight influence on sun protection behaviors.^38^ In our study, even though urban children more frequently practiced sun‐protective measures, their mean daily sun exposure was higher than that of rural children. This could be related to the attitudes of their parents towards the beneficial effects of sun exposure such as vitamin D production. In a study by Balato et al., the most common reason that mothers gave for exposing their children to sunlight was that they believed it was beneficial for children's growth.^39^

Among the limitations of the present study, the following can be mentioned. The Covid‐19 pandemic and the resulting quarantine conditions might have led to spending more time at home and limiting recreational activities. In consequence, it may have reduced the need for sun protection among children and families, causing an underestimation of sun protection behaviors. Although due to nationwide vaccination, the restrictions on outdoor activities were relatively reduced. Furthermore, the collected information about the frequency of each sun protection behavior and sunscreen SPF used by children were insufficient and therefore these variables were excluded from statistical analysis.

Despite the aforementioned limitations, our study provides valuable information about the prevalence of sun protection behaviors and the factors influencing them, as there is a limited number of studies regarding sun protection behaviors among Iranian children. Additionally, our findings provide practical information for developing more accurate interventions and training programs aimed at improving sun protection behaviors.

## 5 CONCLUSION

The result of our study demonstrated strong correlations between the use of sun protection methods among children and various factors such as demographic characteristics, families' socioeconomic level, and parents' sun protection behaviors. Thus, it seems that ongoing education in children and families regarding the usage of sun protection methods, as well as utilizing strategies to provide easy access to these methods regardless of their socioeconomic status can contribute to more use of sun protection methods. To better understand the factors that affect sun protection behaviors among children, further studies on this topic are needed.

## AUTHOR CONTRIBUTIONS

**Nikta Nouri**: Data curation; investigation; writing—original draft; writing—review and editing. **Parisa Iravani**: Conceptualization; methodology; project administration; supervision; validation; writing—review and editing. **Bahareh Abtahi‐Naeini**: Conceptualization; investigation; methodology; project administration; supervision; validation; writing—original draft; writing—review and editing.

## CONFLICT OF INTEREST STATEMENT

The authors declare no conflict of interest.

## TRANSPARENCY STATEMENT

The lead author Bahareh Abtahi‐Naeini affirms that this manuscript is an honest, accurate, and transparent account of the study being reported; that no important aspects of the study have been omitted; and that any discrepancies from the study as planned (and, if relevant, registered) have been explained.

## Data Availability

Data from this study are available upon request to the corresponding author.
